# Reconstruction of critically-sized perineal defect with perforator flap puzzle technique: a case report

**DOI:** 10.1080/23320885.2019.1583568

**Published:** 2019-03-01

**Authors:** D. Kosutic, N. Tsapralis, P. Gubbala, M. Smith

**Affiliations:** a Plastic and Reconstructive Surgery Department, The Christie NHS Foundation Trust, Manchester, UK;; b Gynaecological Oncology Surgery Department, The Christie NHS Foundation Trust, Manchester, UK

## Abstract

Extensive defects in perineal reconstruction cannot be effectively reconstructed with only a single perforator flap or other conventional techniques. We present a combination of three different types of flaps including pedicled ALT-rectus-vastus lateralis, gracilis-PAP flap and two IGAP flaps as an alternative option for reconstructing a critically-sized perineal defect.

## Introduction

Reconstruction of extensive defects encompassing pelvic floor, perineum, vulva and bilateral glutaeal areas following resection of recurrent malignancies is particularly challenging in the setting of previous local flaps, extensive scarring, recurrent infections and radiotherapy. Flap selection depends on defect size and shape, donor site availability, patient choice, surgeon expertise, medical co-morbidities and previous treatments [1].

The optimal wound management technique after abdomino-perineal resection has not been yet determined. It is still impossible to establish a single, optimal, evidence-based procedure for the management of pelvic exenteration [2].

A greater appreciation of the goals, drawbacks and progress in perineal wound management after abdominoperineal rectal resection can facilitate the surgeon make better choices for each patient [2]. There is a range of reconstructive options available for perineal reconstruction, from local flaps to free flaps. Progress in perforator flap surgery has been the main recent advance in perineal reconstruction.

Preceding operations and radiotherapy should be evaluated as they may endanger the vascularity of possible flaps. Desired aesthetic and functional outcome in perineal reconstruction necessitates adequate skin cover and well vascularised tissue to fill dead space. Large defects cannot be effectively reconstructed with only a single perforator flap or other conventional techniques such as gracilis muscle flaps, gluteous muscle flaps and rectus abdominis myocutaneous flaps [3]. Therefore, multiple types of flaps can be used simultaneously for reconstruction of large and composite defects in perineal area in order to achieve a tension free closure and a good aesthetic and functional outcome. Though abdominal-based flaps have been used for largest defects in this area routinely, they may not be large enough when defects extend far beyond perineum or their use is limited when several stoma-openings are required like in our case. We present the use of multiple perforator flaps supplied by perforators from different perforasomes, to reconstruct extended defect including vulva, pelvic floor, perineum and bilateral glutaeal areas following resection of recurrent malignancy in the setting of previous surgeries, infections and radiotherapy. The applicability of this technique is limited in extremely demanding perineal reconstruction cases in which the defect cannot be covered with only conventional type of flaps. Therefore, using multiple perforator flaps for the reconstruction of large defects in the perineal area can serve as an effective alternative option.

## Case report

**Figure 1.**
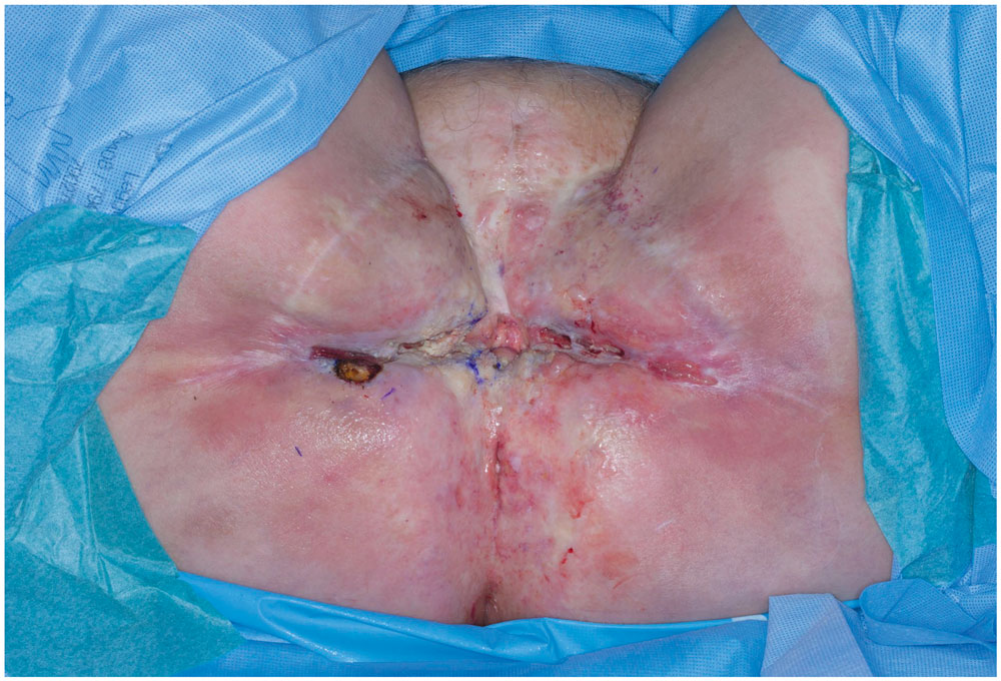
Perineal area prior to resection.

**Figure 2.**
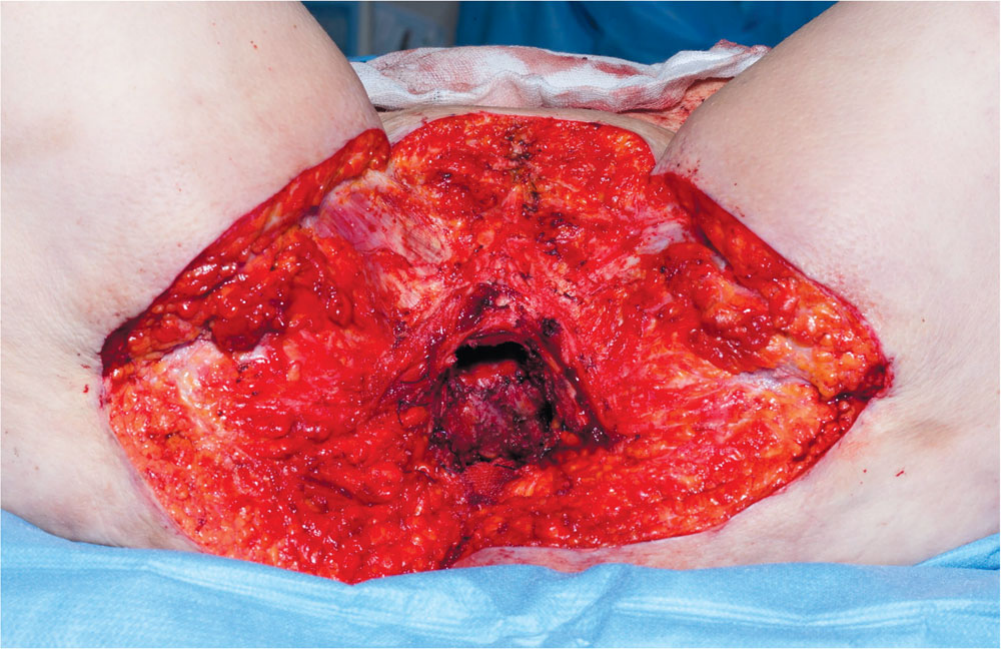
Perineal defect post-resection.

**Figure 3.**
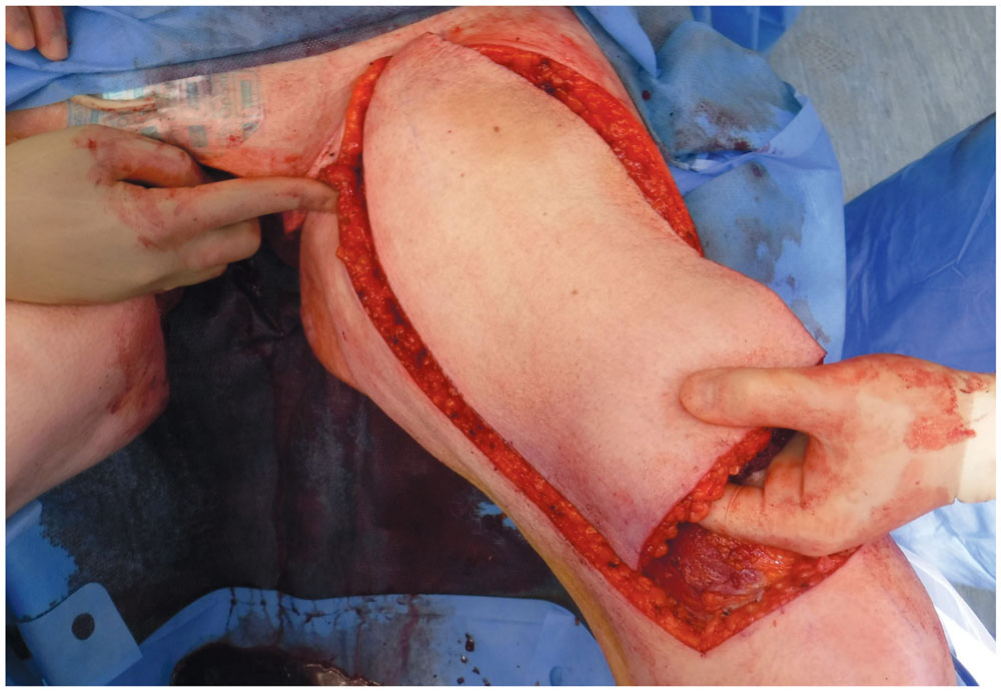
Harvest of Mega chimeric ALT-vastus lateralis.

**Figure 4.**
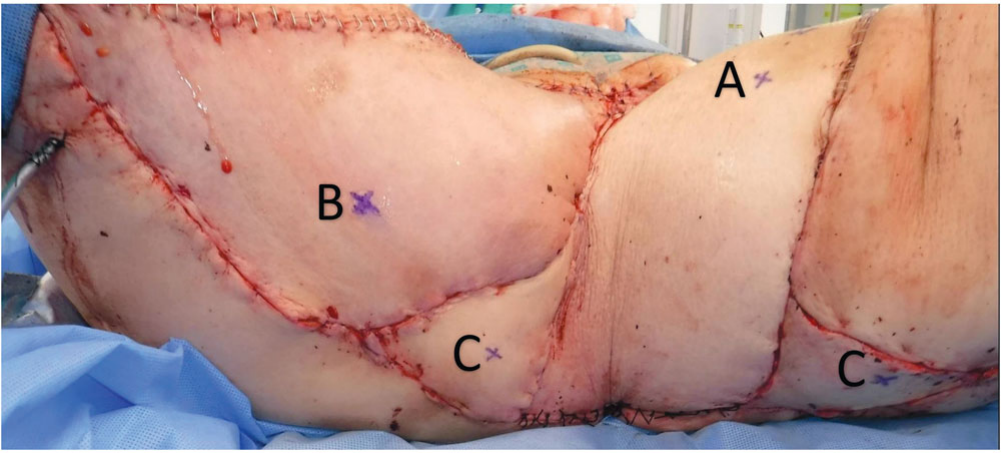
Patient disposed in lithotomy position. A is marked the perforator for the ALT-vastus lateralis, B is marked one of the perforators for the graclis V-Y advancement flap and C are marked the perforators of the two free style perforator flaps.

**Figure 5.**
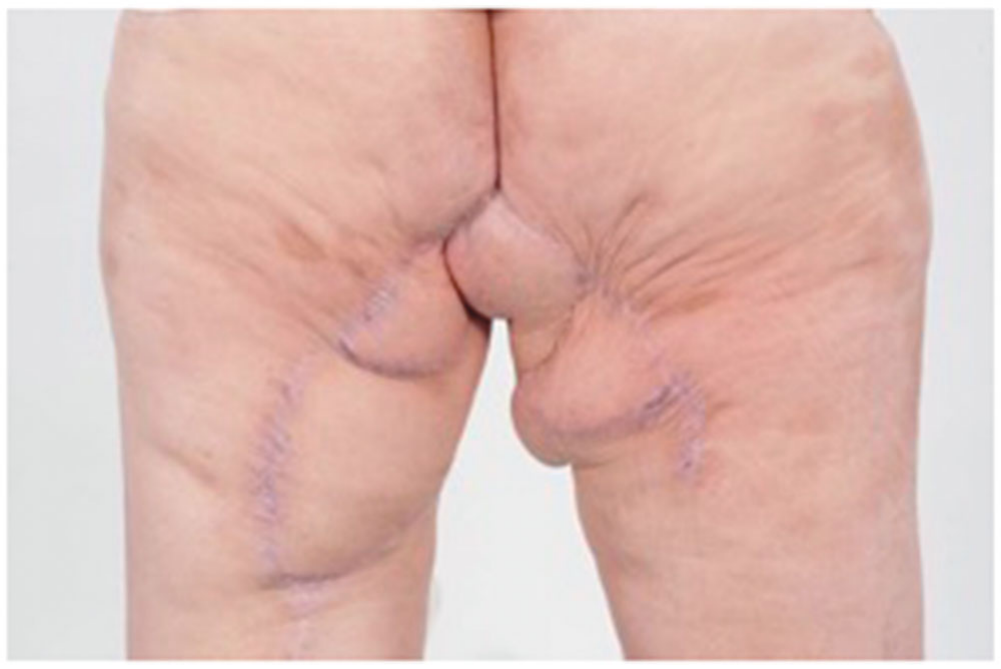
6 months post-operative result.

A 74-year-old woman affected by radio-recurrent squamous cell carcinoma of the vulva, following multiple previous surgeries including V-Y IGAP flap reconstruction, with presence of extensive area of chronically infected, scarred and macerated skin around urethra, anus, perineum and bilateral glutaeal areas (Figure 1) underwent extensive soft tissue resection of these areas alongside pelvic exenteration, colostomy and ureteric stents. Gynecological surgeons performed the resection of uterus and vagina, colorectal surgeons excised rectum, anal canal, urethra, part of the bladder and very large previously irradiated and reconstructed area of soft tissue (Figure 2) followed by colostomy, whilst urologists performed a suprapubic catheterisation in the right side of abdominal wall. Our pre-planned strategy after discussion with Colorectal, Urology and Gynecological surgeons consisted of reconstructing the defect with a pedicled ALT-rectus-vastus lateralis flap and an extended V-Y advancement flap. Unfortunately, the use of the VRAM flap was prohibitive due to colostomy and ureteric stents planned by our colorectal and urology surgeons, who were strongly against the use of abdomen as source of reconstructive options. Our plastic Surgery team consisted of a single surgeon and two assistants and the overall time of the operation was 12 hours. The reconstructive part of the procedure lasted 6 hours. Intra-operative hand-held Doppler was used to identify all perforators in all flaps planned to be used for reconstruction. The extensive pelvic-perineal-vulvar and glutaeal defect was then reconstructed with, a much wider than planned preoperatively, mega-chimeric pedicled ALT-rectus-vastus lateralis perforator flap harvested from the left thigh measuring 31 cm in width and 40 cm in length (Figure 3). The skin and soft tissue in the left groin, between the donor site area and the perineal defect was extensively damaged from previous radiotherapy treatment and discarded. The arc of rotation and advancement of the flap has significantly increased. The flap was then rotated and set into the defect. However, this large flap alone only covered 60% of the total defect surface area. The left thigh donor site required a skin grafting (grafts harvested from the right thigh) and secured with a topical negative wound pressure device. Consecutively, on the contralateral side, we used the hand-held Doppler to identify perforators on the right medial thigh. We identified two perforators - distal and proximal to the defect. Based on these two perforators, we harvested a large chimeric-blood supply extended combined Gracillis-PAP flap to cover a further 30% of the defect in a V-Y advancement fashion. Following this manoeuvre, two smaller areas of the defect remained in the infragluteal region, one on each side, that still required coverage. With the help of hand-held Doppler only, we identified two IGAP-perforators, one on each side, to base two additional small triangular-shaped IGAP flaps (Figure 4). These were harvested in a free-style perforator flap manner. Apart from small area of delayed healing at the flap junctions posteriorly, all flaps healed uneventfully, and patient achieved good and stable functional outcome (Figure 5).

## Discussion

Anatomical and clinical studies of various perforators have been reported, and perforator flap surgery has become a popular method for perineal area reconstruction due to the great number of perforators in the perineal area [4].

Extensive defects encompassing perineum, pelvis, vulva, glutaeal and perianal areas in the setting of extensive scarring, chronic infection, previous local flap reconstructions and radiotherapy are impossible to reconstruct with a single flap. Combining different perforasomes, we can harvest large amounts of healthy soft tissue in spite of the above-mentioned conundrums and achieve good functional outcome. By using robust perforators distributed on the perineal area, we achieved defect coverage but the aesthetics were also satisfactory [5]. The high complication rate of standard techniques such as direct closure has prompted surgeons to look for other options, including the use of conventional myocutaneous flaps to fill the dead space and reconstruct the perineum, and synthetic or biological meshes. The reliability of perforator flaps in perineal reconstructive surgery have led though to more frequent use and appear to have improved outcomes [6].

The most commonly used flap with adequate dimensions to reconstruct extensive three dimensional pelvic and perineal defects is the VRAM or ORAM [7,8]. It is important to assess whether stomas are to be created through the remaining rectus muscle and whether sacrifice may be contraindicated. In this case, abdominal surgeons and urologist colleagues urged us to avoid abdominal-based flaps, due to high risk of a secondary abdominal hernia after the removal of the rectus abdominis muscle.

The chimeric ALT-rectus-vastus pedicled flap is well known for its robust blood supply and excellent arc of rotation but is not often used in perineal reconstruction. However, flap may not be able to reach the most distal part of defect in all patients and may require skin grafting of donor-site, depending on its size.

The gracilis perforator flap takes advantage of the musculocutaneous perforators and permits the transfer of skin and fat overlying the muscle without sacrificing the muscle as a V-Y adipocutaneous advancement flap. In our case two perforators including PAP-one, were identified and preserved in order to maximise vascularity to the large adipocutaneous advancement flap [9,10].

The concept of free style perforator flaps offers a wide freedom in flap selection given the fact that it can be applied where an audible Doppler signal is detected. The free style local perforator flaps can offer wide arc of rotation and it is considered a safe, versatile and reliable surgical technique [11]. The use of inferior gluteal artery perforators in our case was most convenient option due to proximity to the defect.

This a single case report showing that the combination of different type of flaps in unexpectedly extensive perineal defects is necessary. Luckily the perineal area is rich of perforators arising from diverse source vessels. With the use of hand held doppler intraoperatively it is possible to localise these perforators and raise a variety of free style perforator flaps that were not planned preoperatively and ensure well vascularised soft tissue cover of large perineal defects.

To our knowledge, a combination of these three flaps in the reconstruction of perineal region has not yet been reported in the literature. Despite the small postoperative wound dehiscence in the confluence point of flaps posteriorly, the results that we reported were satisfactory from aesthetic and functional point of view. The combination of these flaps represents a valid and reliable option in immediate one stage reconstruction of defects after extensive oncological resection.
